# Emerging 3‑Hydroxy-4-pyridinone Iron Chelates for Iron Deficiency Chlorosis in Soybean: From Molecular Design to Nanofertilizer Delivery

**DOI:** 10.1021/acs.jafc.6c07324

**Published:** 2026-10-23

**Authors:** Simão Pinho, Carla S. Santos, Tânia Moniz, Maria Rangel, Marta Vasconcelos

**Affiliations:** † CBQF − Centro de Biotecnologia e Química Fina, Laboratório Associado, Escola Superior de Biotecnologia, Universidade Católica Portuguesa, Rua de Diogo de Botelho 1327, 4169-005 Porto, Portugal; ‡ LAQV, REQUIMTE, Instituto de Ciências Biomédicas de Abel Salazar, Universidade do Porto, Rua de Jorge Viterbo Ferreira 228, 4050-313 Porto, Portugal; § LAQV, REQUIMTE, Departamento de Química e Bioquímica, Faculdade de Ciências, Universidade do Porto, Rua do Campo Alegre, s/n, 4169-007 Porto, Portugal

**Keywords:** sustainable agriculture, biofortification, fertilizers, Fe-chelates, alkaline soils

## Abstract

Iron deficiency chlorosis (IDC) is a nutritional disorder that adversely affects plants, particularly in calcareous and alkaline soils. The condition arises due to low solubility of iron (Fe) in the soil, which affects effective nutrient uptake and crop yields. The need to address agricultural challenges associated with Fe deficiency, while enhancing crop nutritional value, requires the identification of chelators capable of forming Fe chelates with properties that promote more efficient plant uptake and maintenance of metal homeostasis. Commercial fertilizers suffer from poor biodegradability and potential environmental risks. Chelates derived from 3-hydroxy-4-pyridinones (3,4-HPO) have emerged as a promising alternative, demonstrating increased Fe bioavailability without evidence of persistence in leaching waters. In parallel, delivery systems have gained attention as potential enhancers of nutrient uptake, improving crop yields under alkaline stress, while offering economic and environmental benefits. This Review summarizes the studies regarding the use of 3,4-HPO Fe chelates for IDC correction in soybean crops.

## Graphical abstract

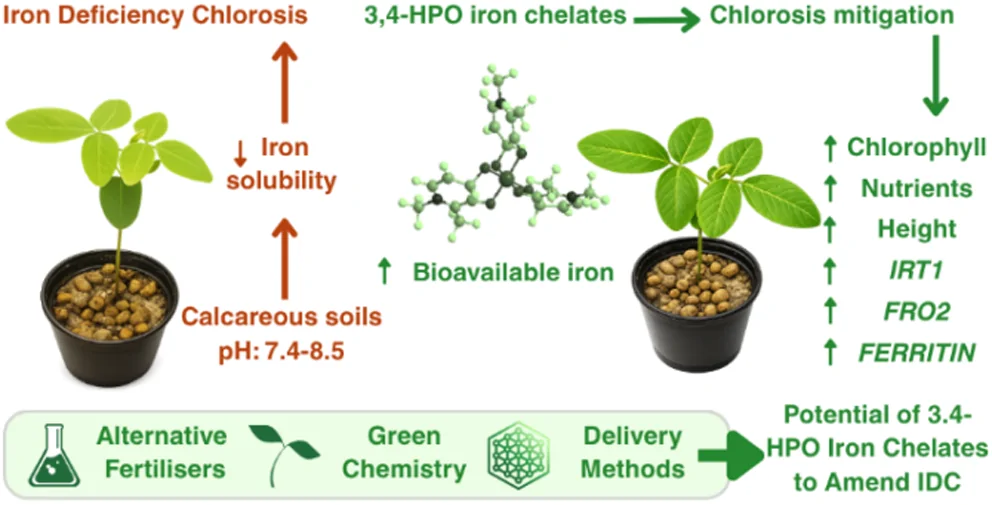

## 1 Introduction

According to the United Nations, the world population is expected to reach nine billion by the end of 2050.^1^ Yet, agricultural yield has not been able to keep up with the increasing food demand, mostly due to water shortages, exhaustion of soil fertility, as well as the impact of climate change, which is expected to intensify the occurrence of extreme weather events, including storms, heat waves, and floods, while further reducing soil nutrients and freshwater availability.^2,3^

As crops constitute one of the main sources of Fe in the human diet, both directly and through animal-derived foods, increasing their Fe content stands as a promising route to reduce human dietary Fe deficiency. The latter affects *ca*. 17% of the global population, especially children, female individuals, and people in low socio-demographic index countries.^4–6^ This type of malnutrition causes severe health complications, including chronic illnesses.^7^ Healthy crops and crop nutritional enhancement can be achieved through different methods, such as plant breeding, genetic engineering, gene editing, and agronomic biofortification.^8^ Several success cases have been reported with these methods.^9^ For example, in India, combined efforts by the International Crops Research Institute for the Semi-Arid Tropics and HarvestPlus were able to successfully use conventional breeding to enhance the levels of iron in pearl millet.^10^

Since most of the iron in the earth’s crust is in an oxidized form that is not easily absorbed by plants, the latter have evolved mechanisms to acquire, process, and store iron.^11^ These mechanisms are classified as Strategy I and Strategy II; the former is responsible for the uptake of ferrous (Fe^2+^) and ferric (Fe^3+^) iron.^11–13^

Strategy I (Figure 1) is usually carried out by higher plants, such as *Arabidopsis thaliana*, potato, and soybean, and consists of three steps.^14,15^ First, plant roots excrete protons via the plasma membrane H^+^-ATPase to lower the pH of the rhizosphere and increase the solubility of Fe^3+^ compounds.^16,17^ Then, a ferric reductase oxidase (FRO) reduces Fe^3+^ to the more water-soluble Fe^2+^, and finally, the element is transported through the cellular membrane with the aid of iron-regulated transporters (IRTs).^14,18^ Other mechanisms include changes in the morphology of the roots, root hair growth, and increments in the concentration of citrates in the phloem.^11,14,17^ The *FRO2* gene plays a central role in producing ferric reductase, along with other genes in the *FRO* family, which have been linked to iron uptake in various plant tissues.^19–21^ The *IRT1* gene codes for a membrane-bound root iron transporter.^22^ Both the expression of *IRT1* and the activity of the reductase are believed to be regulated by the availability of iron within a common regulatory system.^18,19,23^ More recently, coumarins in root exudates have been shown to play an important role in strategy I, with siderins contributing significantly to Fe^3+^ reduction in the rhizosphere in more acidic environments, while fraxetins bind to and mobilize Fe^3+^ for FRO2-mediated reduction in alkaline conditions.^24^

**Figure 1:**
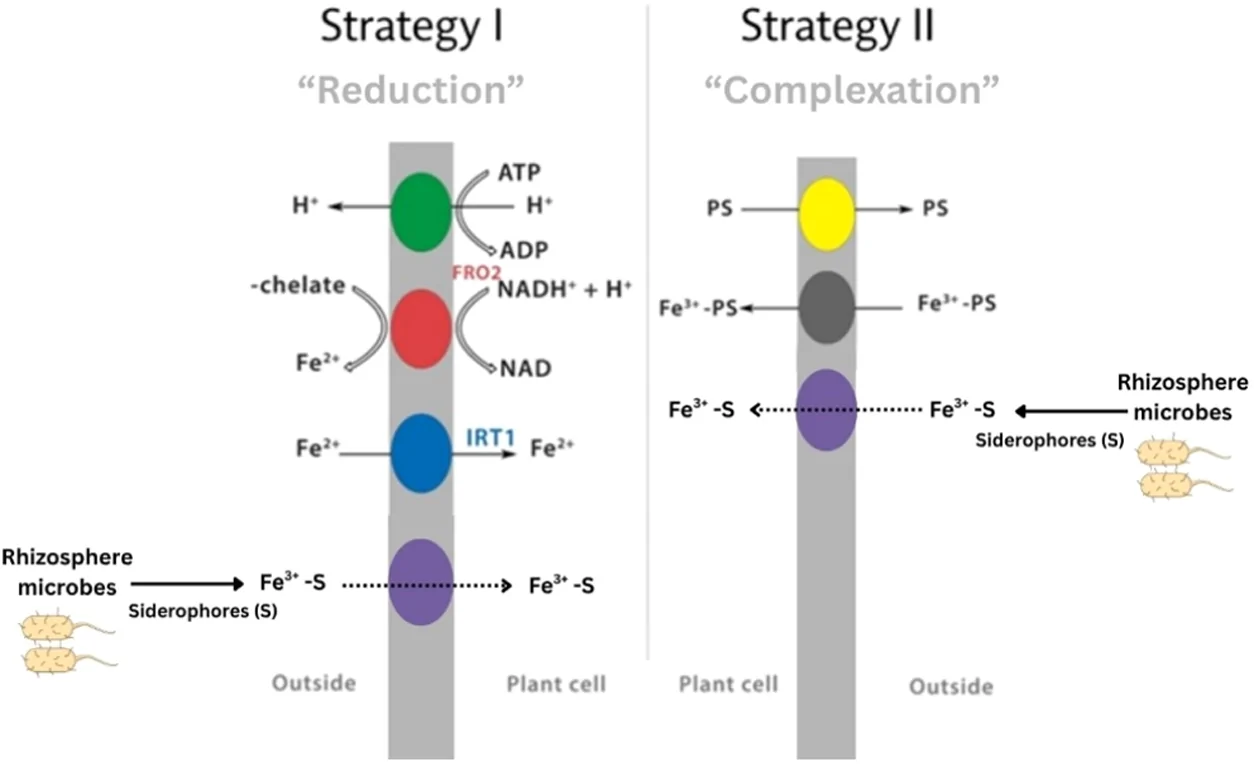
Schematic representation of iron uptake strategies used by plants (Adapted and reprinted with permission from ref 39. Copyright 2026 John Wiley & Sons).

Strategy II plants (Figure 1) include grasses, or gramineous crops, such as corn and wheat.^19,25^ Upon iron deficiency, these plants release phytosiderophores (PSs), which usually are hexadentate chelators with a high affinity for Fe^3+^, that form stable complexes which are then taken up by the roots by a specific transport system.^25–27^ Specific cultivars of certain grass species, such as rice, employ elements of the reduction strategy.^28,29^ They produce lower levels of PS but make up for this by generating metal transporters such as OsIRT1 and OsIRT2 in the root epidermis when facing iron deficiency.^28–30^ Furthermore, ferric chelate reductases belonging to the FRO family have been identified in the roots of Fe-deficient rice plants, further supporting the hypothesis that some species may employ a combined Fe acquisition strategy.^31^

Additionally, there are also microbial mechanisms that impact plant iron uptake. These processes are conducted under low-iron conditions, with some microorganisms in the soil producing high-redox iron carriers that reduce Fe^3+^ to Fe^2+^, and others producing siderophores or organic acids that can mobilize Fe^3+^.^32–34^

Recent review studies further highlight the importance of these processes. For instance,^15^ provide an integrated overview of how microbial siderophores contribute to plant iron nutrition and propose an expanded conceptual framework for plant–microbe interactions in iron acquisition. Similarly,^35^ review the mechanisms of siderophore production by rhizosphere microorganisms and their role in improving iron availability and plant growth under iron-limiting conditions. More broadly,^36^ summarizes the diversity, biosynthesis, and uptake pathways of bacterial siderophores, offering a comprehensive perspective on microbial iron acquisition strategies relevant across environmental contexts.

Following the release of microbial siderophore–Fe complexes into the rhizosphere, plants are generally thought to acquire Fe through their respective uptake strategies.^37^ However, some researchers suggest that some plant species may also take up microbial siderophore–Fe complexes through different transporters.^38^ Despite these findings, the mechanisms underlying the uptake and utilization of microbe-derived Fe complexes require further investigation.

After root uptake, since aqueous Fe^3+^ has low solubility and potential toxicity, the element is complexed by chelating agents, like citrate, to enable its transport throughout the plant.^11^ The toxicity of aqueous Fe is related to its ability to redox cycle, which may lead to the production of oxygen free radicals, harming many cell structures, and leading to cell death in the long run.^40,41^ The toxicity of Fe is diminished if Fe is complexed with available ligands, since the redox potential is fine-tuned by the ligands that chelate the element. Nicotianamine (NA), a nonproteogenic amino acid, is responsible for the chelation of iron in the phloem, while in the xylem, this function is mainly performed by citrate.^42,43^ A class of specific transporters of the Yellow Stripe-Like (YSL) family is responsible for the translocation of iron from the xylem to the phloem, while also being crucial for the absorption of Fe^3+^-PS complexes at the soil-root interface in Strategy II plants.^42,44^ The storage and buffering of iron at the plants’ cellular level allow them to cope with its toxicity and scarcity, and this function is performed by organelles such as plastids and vacuoles, where ferritins store a large fraction of cellular iron and maintain homeostasis.^42,45^

Mainly located within the chloroplasts, ferritins are a class of iron-storage proteins responsible for maintaining Fe homeostasis, comprising hollow spheres composed of 24 subunits that may contain 4500 iron atoms per molecule.^46–48^ These proteins are encoded by the *FERRITIN* gene family, which is upregulated by iron.^49,50^ The influx of Fe molecules into these organelles, in both strategy I and strategy II plants, is thought to require a reduction-based strategy, made possible by a member of the FRO family, FRO7, responsible for converting Fe from the ferric to ferrous form in order to enter the chloroplast.^30,51^ Ferritin synthesis is also heavily regulated by the amount of Fe inside the cells, increasing in high amounts when there is an excess of Fe, as a way to contain the element and prevent the formation of reactive oxygen species.^46,50,52^ This regulation involves a large number of proteins, including the basic Helix–Loop–Helix (bHLH) protein family, which comprises at least 17 regulatory factors.^53^ In soybean root tissue, two transcription factors of this family, namely bHLH57 and bHLH300, whose production is upregulated by Fe deficiency, induce the expression of *IRT1* and *FRO2* genes when both proteins are present.^54^

Due to its importance as part of various proteins and enzymes, Fe plays a crucial role in basic biological processes such as photosynthesis, chlorophyll synthesis, respiration, nitrogen fixation, uptake mechanisms, and DNA synthesis, as well as being an active cofactor of many enzymes responsible for plant hormone synthesis.^40,55,56^ In the photosynthetic framework, there are 12 Fe atoms in photosystem I, two or three in photosystem II, and five in the cytochrome complex, resulting in a sharp decrease in photosynthetic activity under Fe deprivation.^57,58^ Due to their ability to delocalize electron density over both iron and sulfur atoms, iron–sulfur [Fe–S] clusters are essential constituents in the respiratory and photosynthetic electron transport chains, where they define the electron transport pathways in many membrane-bound and soluble enzymes and are essential elements in the active centers of ferredoxins, a class of proteins responsible for carrying electrons.^59,60^

When iron absorption is limited, as in alkaline soils, crops may develop iron deficiency chlorosis (IDC), which hampers crop productivity and affects the iron nutritional value of both vegetative tissues and edible plant parts.

## 2 Iron Deficiency Chlorosis and the Impact on *Glycine max* L. Crops

Soybean (*Glycine max* L.) is a dicotyledonous, nongramineous plant, part of the *Fabaceae* family. Soybean seeds have been shown to be a rich source of protein, comprising 34% to 57% of their dry weight and containing at least eight essential amino acids.^61–63^ They are also rich in oil, with dry seeds ranging from around 4% to 24% of their weight, depending upon the variety, with this amount being negatively correlated with the total protein quantity.^64–66^ Soybeans also contain high dietary fiber, between 15% and 24%.^67–69^ These legumes are also rich in minerals such as magnesium, zinc, calcium, and iron.^70^

A recent study showed that consuming 50 g of soybean products can provide *ca*. 37% and 32% of the daily iron intake recommended by the Spanish Agency for Food Safety and Nutrition, for men and women, respectively.^71^ Although promising, these results do not account for the fact that, in soybean, Fe is in a nonheme form, which has lower solubility and bioavailability than heme iron.^72,73^ Nevertheless, soybeans have considerable potential as a dietary source of iron.^74^ Given that soy-based food products are an important protein source for vegan and vegetarian populations—groups that are more vulnerable to iron deficiency,^75,76^ their capacity to provide meaningful amounts of iron becomes particularly important.

In the past 60 years, the global production of soybean has risen rapidly, having increased from 20 to 30 million tonnes per year in the 1960s to nearly 400 million tonnes in 2024 (Figure 2), with projections exceeding 420 million tonnes in the coming years.^77,78^ The two main soybean-producing countries are the United States of America and Brazil, which altogether produce about 69% of the world’s total yield.^78,79^ This crop has become one of the most produced agricultural products in the world, not only due to the qualities previously mentioned, but also other components, such as phospholipids, vitamins, minerals, and some other minor substances such as trypsin inhibitors, phytates, oligosaccharides, and isoflavones.^80^ The majority of global soybean production is directed toward animal feed, and only about 20% of all output is converted into food products such as edible oil, soybean milk and tofu, destined for human consumption.^81^ Soybean oil is the second most produced vegetable oil in the world, and most of it is used in food manufacturing and as a biofuel.^82–84^ As of 2023, soybeans represent the most heavily traded agricultural commodity globally, ranking fourth among all commodities.^81^ The gross production value of soybeans has also increased rapidly since 2000, having reached around 195 billion US$ in 2023.^77^

**Figure 2:**
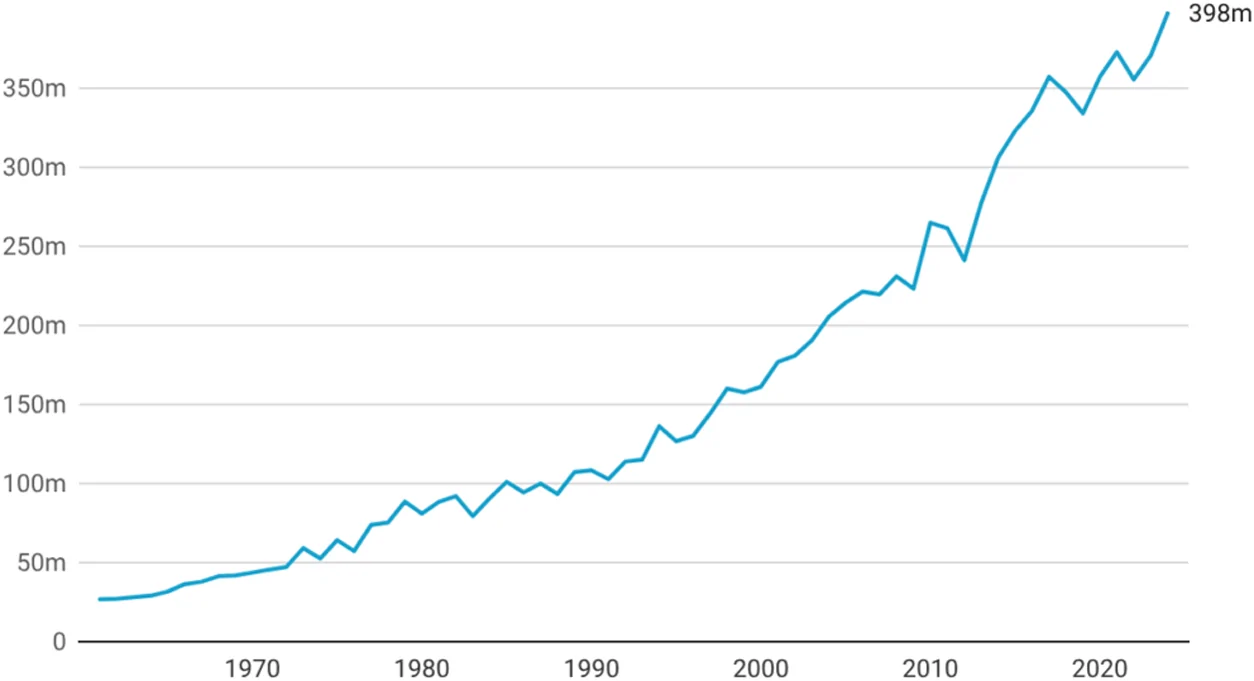
Global soybean production from 1961 to 2024. Values on the *y*-axis are expressed in millions (m) of tonnes.^77^

In the European Union, a 2018 report from the European Commission has reinforced the importance of reducing the dependency on plant proteins imported from the Americas, with the intent of increasing self-reliance, encouraging more balanced diets within the EU countries, and reducing deforestation in South America.^85,86^ The European Green Deal (2019) reinforced this trajectory by recognizing legumes, including soybeans, as relevant components of the agroecological transition and of efforts to reduce the environmental footprint of European agricultural activity, particularly through the Farm to Fork Strategy.^87,88^ On July 17th of 2017, 14 Member States endorsed the ″European Soya Declaration”, pledging their support for the promotion of sustainable and certified production and marketing of legumes within Europe.^89^ Following this growing demand and encouragement, particularly for locally sourced and non-genetically modified soybeans, the dedicated cultivation areas in Europe (EU-27) have seen a rapid expansion in recent years, increasing from 430 000 hectares of area harvested in 2010 to more than a million hectares in 2022, with the main producers being France, Italy, and Romania.^77,85^

IDC is a significant nutritional disorder affecting plants, particularly *Glycine max* L., cultivated on calcareous soils (pH 7.4–8.5), where elevated pH values reduce iron bioavailability in the rhizosphere, ultimately impairing its uptake, transport, and physiological utilization.^90^ This condition is visible through yellowing on the upper leaves, interveinal chlorosis, and stunted growth, leading to a decline in yield and quality up to 25% in soybeans, causing extensive monetary expenses, especially since around 30% of the world’s total arable land is composed of calcareous soils.^90–92^

The causes of IDC are mostly related to cultivation in soils that present high pH values. In this context, iron in the soil tends to exist in the ferric state (Fe^3+^), mainly in the form of very insoluble oxides and hydroxides, making it unavailable for uptake and transport in strategy I plants.^93^ Calcareous soils are also known to possess high amounts of calcium carbonate, which, in conditions of high moisture, is dissolved, forming a solution containing calcium (Ca^2+^) and carbonate (CO_3_^2–^) ions, thus increasing the pH of soils, further oxidizing Fe^2+^ to Fe^3+^ and acting as a buffer, preventing the strategy I plants from lowering the soils’ pH.^93^

Commonly, IDC’s visual symptoms are measured on a scale of 1–5 (Figure 3), in which 1 represents no symptoms, 2 indicates some chlorosis on the canopy, 3 indicates a higher degree of chlorosis, 4 implies the existence of some necrosis, and 5 represents severe chlorosis and necrosis; however, this visual rating system is quite subjective.^93,94^

**Figure 3:**
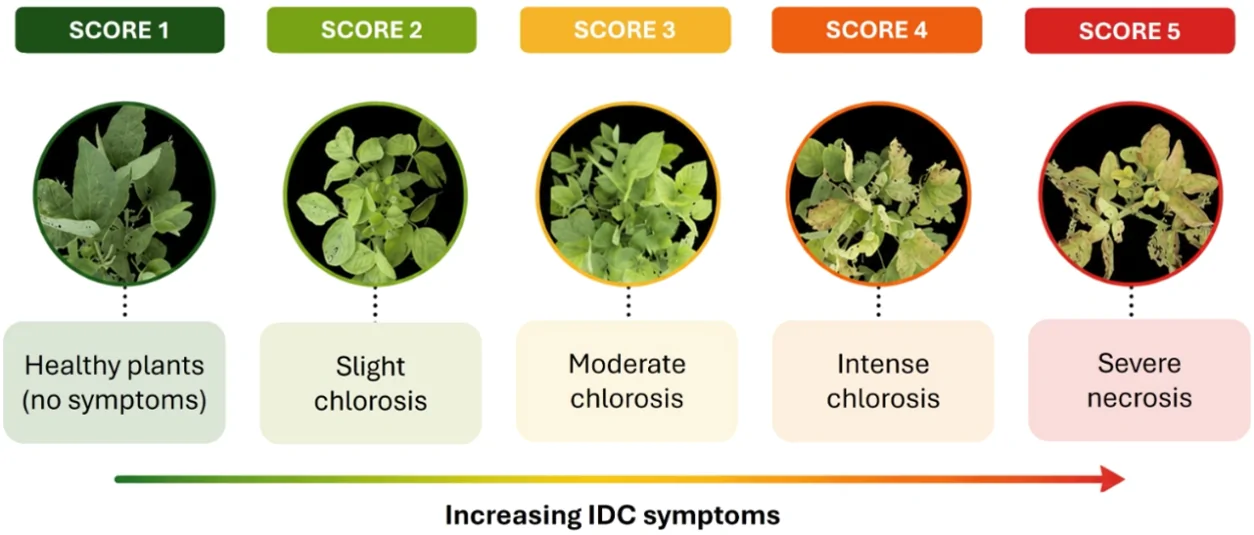
Iron chlorosis visual rating scale representation. Adapted and reprinted with permission from ref 95. Copyright 2026 Springer Nature.

The early vegetative stages of soybean begin with emergence, where the seed absorbs water to initiate germination. This is followed by the cotyledon stage, during which the first leaves expand, and the cotyledons provide the nutrients. In the subsequent stages, the plant consecutively develops its first (V1), second (V2)—where nitrogen fixation begins—and third (V3) set of trifoliate leaves, typically reaching a height of 20 cm. Symptoms of IDC usually appear during these early stages.^96^

As a strategy I plant, soybean is susceptible to IDC, primarily due to its sensitivity to alkaline stress. A survey conducted in 2003 revealed that soybean farmers in the United States of America (the world’s largest soybean producer at that time) attributed 24% of their crop yield loss to IDC, despite using IDC-resistant genotypes.^97,98^

## 3 Iron Deficiency Chlorosis and Mitigation Strategies

Conventional plant breeding is one of the techniques to reduce the prevalence of mineral deficiency, drought, and other abiotic stresses.^8^ Genetic engineering and gene editing are other approaches that can be used to prevent or reduce the symptoms of IDC.^8,99^ A well-known example is the overexpression of *AtFRO2* from *Arabidopsis thaliana* in soybean, which significantly increased ferric chelate reductase activity and Fe accumulation in tissues but did not improve tolerance to IDC in alkaline soils, indicating that Fe^3+^ reduction is not the main limiting factor.^100,101^ Other studies show that improved tolerance also depends on Fe transport systems such as IRT1, rhizosphere acidification via H^+^-ATPases, and transcriptional regulators like FIT and bHLH that coordinate Fe deficiency responses (reviewed in ref 102). In soybean, genes such as *Gm03* and *Gm20* have also been associated with IDC tolerance.^103^ Another potential way to reduce IDC is soil acidification, which can enhance Fe bioavailability. Overall, effective strategies require multifactorial approaches.

Nevertheless, the application of different types of Fe-based fertilizers is probably the most used method to combat IDC, consisting of Fe complexes of ligands with an appropriate affinity for iron, high solubility in water, and bioavailability.^8,91^ The three main types of iron fertilizers are inorganic iron salts, which are relatively inefficient in the soil, since they turn into insoluble compounds, being usually applied as foliar fertilizers; iron complexes obtained from natural sources, such as those derived from humic acids, which are also quite unstable in the soil and usually applied in the same manner; and synthetic iron chelates, which possess ligands that provide the required properties for either foliar or soil application.^104,105^

Several Fe^3+^ chelates of ligands such as ethylenediaminetetraacetic acid (EDTA), ethylenediamine-*N,N*’-bis­(o-hydroxyphenylacetic) acid (EDDHA) and diethylenetriaminepentaacetic acid (DTPA) have all been used in the soil as a way to provide the necessary supply of bioavailable Fe; however, like many other commercial fertilizers, these compounds have been shown to be extremely resistant to biodegradation and can cause severe damage to the environment by binding to other heavy metals and potentially leaking to groundwater.^106–109^

In response to these limitations, recent research has explored a wider range of ligands for Fe chelate fertilizer development, aiming to try to find novel compounds with improved biodegradability and high agronomic efficiency. Table 1 summarizes selected commercially used and alternative ligands and their key performance characteristics.

**Table 1:** Overview of Aminopolycarboxylates with Low Biodegradability and Biodegradable Ligands Used as Fe-Chelate Fertilizers

Type of Ligand	Ligand	Performance and Environmental Profile	Type of Application	Reference
Commercial aminopolycarboxylates with low biodegradability	EDTA (ethylenediaminetetraacetic acid)	Across multiple crops and pH ranges, improves iron bioavailability, crop yield, and plant nutritional quality. Most used chelate for agricultural applications. Low biodegradability.	Soil and foliar application/seed treatment/soilless cultivation	^110–112^
	EDDHA (ethylenediamine-*N,N*′-bis(2-hydroxyphenylacetic acid))	Across multiple crops, improves bioavailability, crop yield, and plant nutritional quality. Performs well in alkaline soils. Widely used. Low biodegradability.	Soil and foliar application/seed treatment/soilless cultivation	^110,112,113^
	DTPA (diethylenetriaminepentaacetic acid)	Across multiple crops, improves bioavailability, crop yield, and plant nutritional quality. Performs well in acidic soils. Widely used. Low biodegradability.	Soil and foliar application/seed treatment/soilless cultivation	^110,114,115^
Biodegradable chelators	IDHA (*N*-(1,2-dicarboxyethyl)-d,l-aspartic acid)	Across multiple crops, demonstrates limited agronomic effectiveness in soil due to low stability and rapid degradation; generally, less effective than EDDHA/EDTA; high biodegradability restricts persistence.	Soilless cultures/foliar application	^90^
	[*S,Ś*]EDDS (ethylenediaminedisuccinic acid)			
	IGSA (2-((1,2-dicarboxyethyl)amino)pentanedioic acid)	In a study with lettuce (Lactuca sativa), enhances biomass production and mineral uptake; outperforms EDTA and shows competitive performance relative to EDDHA; high biodegradability.	Soil and foliar application	^116^
	GLDA (glutamic-*N,N-*diacetic acid)	In a study with lettuce (Lactuca sativa), it exhibits high biodegradation (>60% within 28 days) and promotes significant increases in plant biomass and growth; lower complex stability than EDTA but reduced environmental persistence and accumulation risk	Hydroponics and soil application (root and foliar treatment)	^117^
	lignosulfonate (LS)	In a study with soybean (Glycine max), shows lower Fe mobilization and plant uptake efficiency compared to EDDHA and EDTA; moderate biodegradability.	Soil application	^118^
	gluconate (GA)			
	plant-derived peptide biochelates	Across multiple crop systems, achieves Fe supply efficiency comparable to EDDHA and EDTA; high biodegradation with 69–82% reduction in environmental burden.	Hydroponics	^119,120^
	amino acid chelates	Across multiple crop systems, improve chlorophyll content, seed weight, and protein accumulation compared to EDTA; high biodegradability but lower complex stability than EDDHA.	Hydroponics	^121,122^
	HIDS (hydroxy-iminodisuccinic acid)	In a study with rice (Oryza sativa L.), it improved iron bioavailability. In another study with radish (Raphanus sativus), improved growth. In both cases, they had better results than EDTA. High biodegradability.	Soilless cultures	^123,124^

Since both FeEDTA and FeEDDHA are currently top performers in the market, it is crucial to explore other classes of chelators that can minimize the environmental impact of Fe fertilization in soybean plants without significantly sacrificing efficiency, ensuring that the agricultural practices remain both productive and eco-friendly.^106,125^ An older study that compared the effects of FeEDTA as a foliar fertilizer, FeEDDHA as a seed coating agent, and IDC-resistant cultivars to mitigate IDC concluded that using IDC-resistant cultivars is a more efficient approach.^126^

## 4 Fe Chelates of 3-Hydroxy-4-pyridinone Ligands as Alternative Fertilizers for Soybean Crops

Over the past decades, several alternative fertilizer formulations have been investigated to enhance Fe delivery while reducing environmental impacts, as summarized in Table 1. Many have been abandoned for various reasons, such as being animal carcinogens or being potentially toxic to plants.^127–129^ Ten years ago, our groups proposed 3-hydroxy-4-pyridinone (3,4-HPO) ligands (Figure 4A) as a new class to produce Fe fertilizers. The innovation and key idea of this proposal are the formulation of Fe-chelates based on a distinctive family of chelators that allows the design of compounds with a variety of chemical properties that can be fine-tuned according to the results obtained from the evaluation of their biological properties. Such a possibility allows not only the improvement of the performance of the fertilizer but also the investigation of the mechanisms underlying their activity.

**Figure 4:**
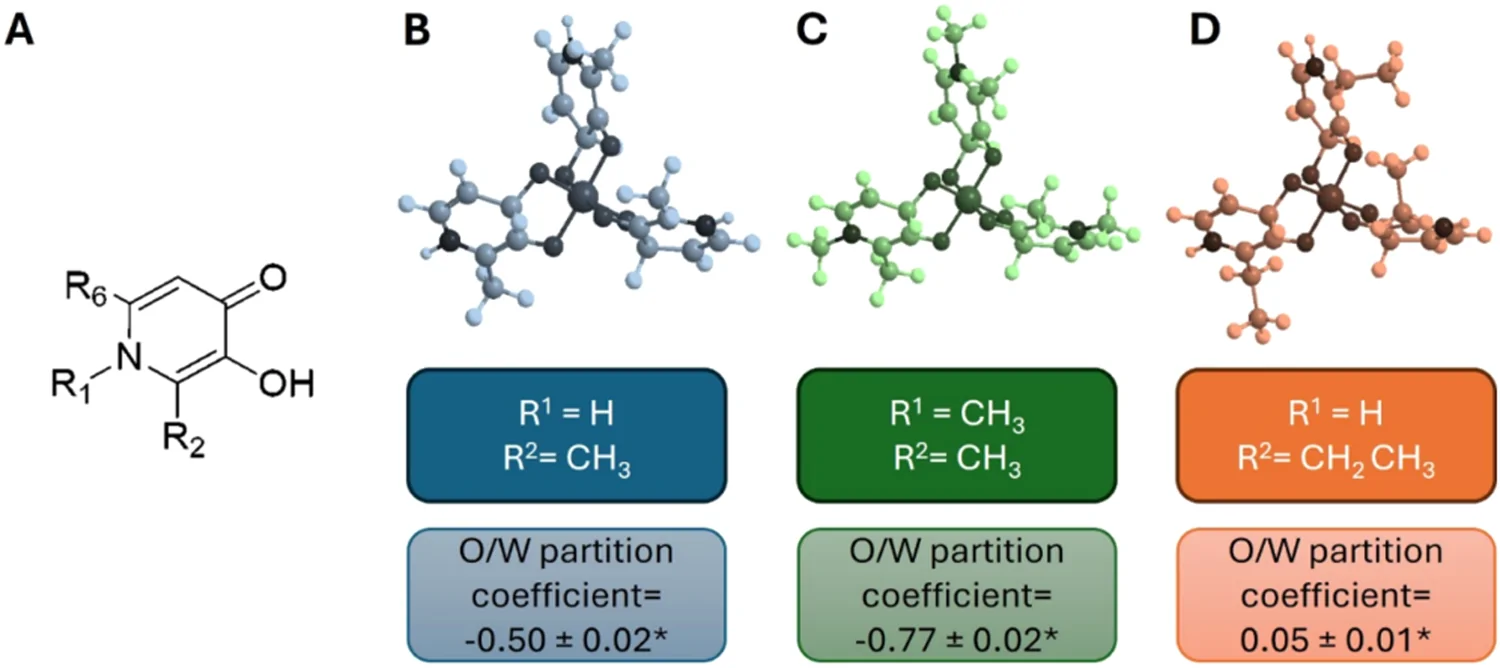
General chemical formulas of 3,4-HPO (A) and ligand substituents and log D values of three different 3-hydroxy-4-pyridinones used to synthesize the iron chelates used as fertilizers (B—[Fe­(mpp)^3^], C—[Fe­(dmpp)^3^], D—[Fe­(etpp)^3^]) (adapted from ref 132). R^1^ and R^2^ refer to the chemical groups present in positions 1 and 2 of the heterocyclic ring, respectively; R^6^=H. * 1-Octanol/water (O/W) partition coefficient calculated for each ligand in refs 150,151 .^150,151^

The idea emerged since chelates of the 3,4-HPO family have already attracted attention for their clinical use in the treatment of Fe overload in β-thalassemia patients.^130,131^ These ligands are advantageous due to their very high affinity toward M­(II) and M­(III) metal ions, their synthetic versatility, and the possibility to adjust some physicochemical properties without significantly changing the affinity of these chelates toward specific metal ions or the redox potential of the metal ion complexes.^132,133^

Ligands of the 3,4-HPO type are bidentate oxygen ligands, thus providing a 6-oxygen coordination sphere in the *tris* complexes of M­(III) metal ions, [ML_3_], and a 4-oxygen coordination sphere in the *bis* complexes of M­(II) metal ions, [ML_2_]. The values of the stability constants of the complexes formed by 3,4-HPOs are quite large and are reported in the literature.^134–136^ The values measured for Fe^3+^and Fe^2+^ complexes, by potentiometric and spectrophotometric methods, made from a variety of ligands lie in the range 35.0 < log β_3_ < 37.0 and 12.0 < log β_2_ < 15.0. Since data regarding stability constants refer to standard thermodynamic conditions, under biological conditions, it is usual to refer to pFe values (defined as the negative logarithm of the free Fe^3+^ (aq) concentration calculated at pH 7.4), with a total ligand concentration of 10 μM and [Fe^3+^ aq]_t_ of 1 μM. The pFe values for 3,4-HPO lie in the range 19.4 < pFe < 21.5,^137^ which are very satisfactory for bidentate ligands.

The stability constants of Fe^3+^ complexes with the hexadentate polyaminocarboxylate ligands used as fertilizers are in the range 25.0 < log β_3_ < 35.0.^138–140^ Yunta et al. calculated values of pFe for EDDHA in several pH conditions in water and also in agronomic conditions, and the values range within 22.1–25.9 for pH 7 and 15.0–15.28 in agronomic conditions.^138^

Considering the above information and the speciation at pH = 5.5 reported by us,^91^ we believe that the set of chelators we proposed as potential alternative fertilizers fulfill the requirements regarding stability constants and pFe values. For a hexadentate 3,4-HPO ligand prepared in our lab,^141–143^ the values are log β = 34.40 and pFe = 29.84, thus reflecting the influence of the 1:1 stoichiometry and entropy contributions.

3,4-HPO ligands are amphiphilic, as they may contain both hydrophilic and hydrophobic groups, granting them the property of hydrophilic–lipophilic balance.^132,136^ Moreover, amphiphilic character is crucial for the interaction of the molecule with lipid bilayers and consequently for its affinity, permeation properties, and preferential location within membranes. The latter properties are determinant for the chosen molecular pathways within the cell and molecular interaction with protein membranes, namely, transporters and receptors.

Examples of the importance of the amphiphilic character of 3,4-HPO for different applications in the environmental, biomedical, and agricultural fields have been reported.^144–146^

At present, several studies corroborate the idea that Fe chelates of the 3,4-HPO family have shown their ability to increase iron bioavailability in soybean plants.^91,132,147^

The first studies using [Fe­(mpp)_3_] (Figure 4B) showed it outperformed FeEDDHA in hydroponically grown soybean, increasing biomass and iron accumulation by ∼50%, without inducing iron-stress genes *FRO2* and *IRT1*.^104^ The lack of induction of these Fe-deficiency markers suggests that Fe was readily available to the plants following treatment with [Fe­(mpp)_3_], preventing the activation of the typical Fe-deficiency response mechanisms associated with these genes.

Subsequent work compared [Fe­(mpp)_3_], [Fe­(dmpp)_3_], and [Fe­(etpp)_3_] (Figure 4B,C, respectively) under varying pH and concentrations, revealing that [Fe­(mpp)_3_] was most effective, likely due to its favorable hydrophilic–lipophilic distribution in membranes.^132^ Moreover, the results show that the compounds exhibit a differential distribution of accumulated Fe in the roots and the leaves, which is also in agreement with the hydrophilic–lipophilic balance. A biophysical study using labeled liposomes prepared from a soybean lipid extract revealed that the distribution of the compounds on model membranes is in agreement with their structure and that the most effective chelate, [Fe­(mpp)_3_], has a preferential location close to the surface, while the others prefer the hydrophobic region inside the bilayer.^132^ We hypothesize that compounds may follow different pathways within the plants, thus providing an explanation for the differences in Fe distribution. Also, preferential location at the surface may favor uptake by the reduction strategy. Although the exact uptake pathway of 3,4-HPO chelates remains unclear, differences in hydrophilic–lipophilic balance appear to influence their interaction with biological membranes and may contribute to the distinct Fe distribution patterns observed in soybean plants. In particular, [Fe­(mpp)_3_] promotes greater Fe accumulation in shoots, whereas [Fe­(dmpp)_3_] tends to favor Fe retention in roots, suggesting that membrane interaction may be an important determinant of chelate efficiency.^91,132^

The studies performed for chelate [Fe­(mpp)_3_] at variable concentrations and pH values of the hydroponic culture medium demonstrate that the compound may be an efficient Fe fertilizer, even at half the concentration as those previously used for 3,4-HPO chelates and commercial fertilizers.^91^ Chelate [Fe­(mpp)_3_] showed a good efficacy under pH conditions simulating an alkaline soil, even showing a better efficiency in providing Fe to plants, as the increased Fe contents demonstrate.

Field and soil studies confirmed that foliar application of [Fe­(mpp)_3_] improved chlorophyll and nutrient uptake and mitigated IDC symptoms more effectively than FeEDDHA, with no adverse effects detected under the conditions evaluated so far.^147–149^ Seed coating experiments showed minor differences, but overall, [Fe­(mpp)_3_] proved to be a promising iron fertilizer for soybean under alkaline conditions.

Importantly, all studies performed so far with 3,4-HPO chelates have been conducted in soybean, a strategy I species. Therefore, current evidence for their efficacy is limited to reduction-based Fe acquisition systems, while their performance in strategy II species remains unknown.

Another possible way to decrease the environmental impact of agrochemical usage is to improve the efficiency of their uptake, which has brought interest to novel delivery methods, one of them being through nanotechnology.^152^

## 5 Advanced Trends for Fertilization: The Use of Nanoparticles as Delivery Strategies

Nanoparticles (NPs) are often defined as particles in the range from 1 to 100 nm in at least one dimension, although some accept this limit extending up to 1000 nm.^153,154^ The small size of these particles, as well as their large surface area, high particle number per mass unit, and the fact that the atoms on the surface have fewer direct neighbors, grant NPs special properties relating to their ability to successfully interact with their surrounding environment compared to particles inside the bulk solid of the same material, since they tend to be more affected by the behavior of the atoms or molecules that comprise them.^155,156^ Such features may allow for a more efficient nutrient delivery than conventional fertilizers,^157^ as extensively reviewed by Zhang and colleagues.^158^

Nanoparticles can be used as efficient nanocarriers for delivering bioactive compounds, such as pharmaceutical drugs or agrochemical agents, designed to provide a targeted delivery of a required dose in a more time-controlled and eco-friendly manner, as well as protect the therapeutic loads and help them overcome biological barriers.^159,160^ They are usually classified based on their composition, namely, organic, carbon-based, and inorganic NPs, as depicted in Figure 5, and reviewed in many papers.^155,156,159,161^

**Figure 5:**
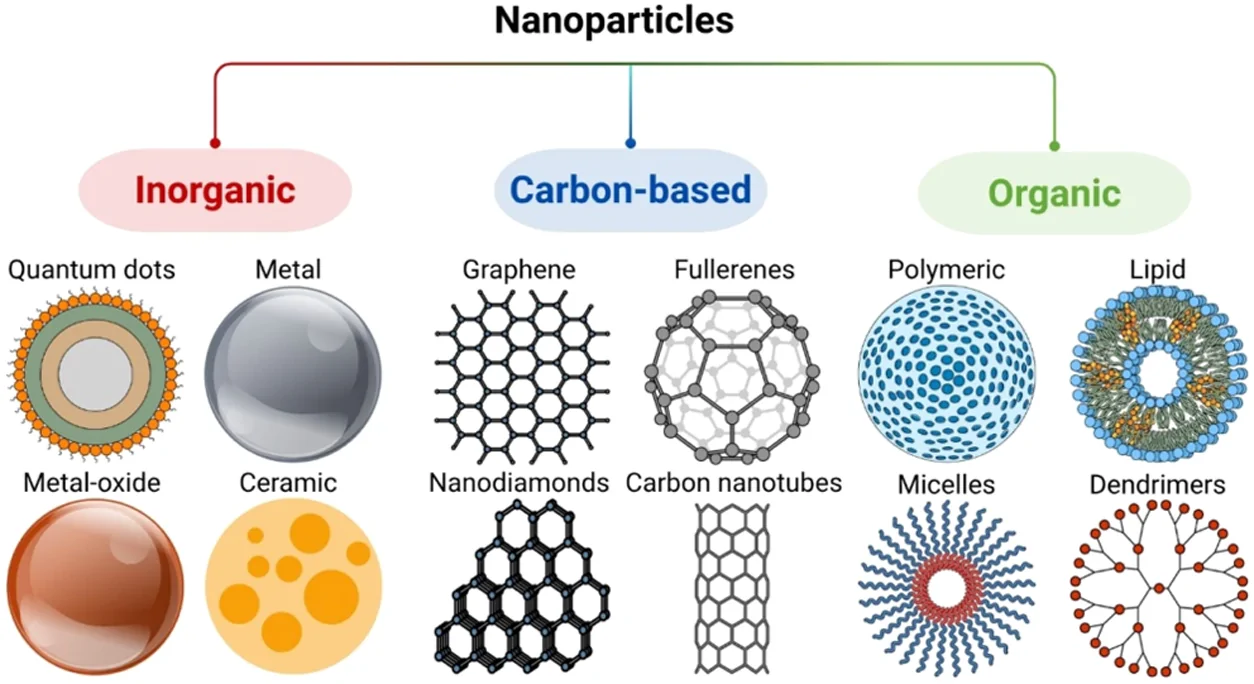
Simplified representation of the predominant types of nanoparticles.^162,163^

Many studies have used NPs for the delivery of fertilizers, proving that the uptake and interaction of nutrients can be augmented, concomitantly increasing crop yield, therefore offering relevant economic and environmental advantages.^154,164^ This efficiency is exacerbated by the fact that plant roots, leaves, as well as some seeds, are porous on the nano level, allowing for NPs to enter the plant more easily, either via root or foliar application.^165,166^

One of the main issues with the current method of application of chemical fertilizers is that a considerable portion of these materials is not absorbed by crops, remaining instead on the soil, where they may eventually be washed away by water, either horizontally or vertically to the groundwater, or go into the atmosphere by volatilization.^159^ This inefficiency is particularly pronounced for Fe fertilization, where plant uptake is typically very limited, with Fe being immobilized in the soil and thus largely unavailable to crops.^167^ By utilizing nanocarriers, the uptake and interaction of nutrients can potentially be augmented, thus pointing out the advantage of nanotechnological approaches.^168^

The uptake of NPs inside plants is highly dependent on various factors, such as plant physiology, type of application, NP size, surface charge, and shape.^169,170^ For root uptake, NPs are initially adsorbed to the root surface, either on the primary or secondary root epidermis, where they can cross it via small pores in the root cell wall or pass through the intercellular space without crossing the cell membrane.^171,172^ Some studies have shown that while positively charged NPs have a greater affinity for the negatively charged outside layer of root epidermis, they are more likely to remain adsorbed to the roots, while negatively charged particles are more likely to be translocated into the apoplast.^173,174^ In order for uptake and translocation to occur, NPs have to enter through certain root cuticles and go through the epidermis, cortex, endodermis, and finally cross the Casparian strip into the xylem, in order to be transported upward into the shoots.^175,176^ These cuticles, usually on root hairs or growing root tips, are usually underdeveloped and permeable, and NPs may be directly exposed to the epidermis, where they are hypothesized to take either the apoplastic pathway, in which NPs penetrate the space between the cell walls or pass through the intercellular space, or the symplastic pathway, which consists of cell-to-cell transport.^177,178^ In the apoplastic pathway, the particles are then allowed to pass through the cortex endodermis, but the Casparian strip has to be bypassed in specific zones where it is disconnected.^179^ In the symplastic pathway, it is hypothesized that NPs may cross plant cells through channels called plasmodesmata.^180^ After reaching the xylem, NPs can then be transported upward to the shoots.^181^

For foliar uptake, once NPs are sprayed onto the leaves, they deposit on the leaf surface, where they are usually absorbed through cuticles, which only allow NPs in the scale of 5 nm to pass, or through the stomata, which can allow for the entry of hydrophilic NPs up to 100 nm.^171^ The ability of NPs to be absorbed is also dependent on the characteristics of the species of the plant itself, with some studies showing that dicotyledonous plants may be more efficient in taking up NPs.^172^ After entering the leaf, the NPs progress through the previously described apoplastic or symplastic pathways to reach the vascular system, from which their loading into the phloem enables long-distance transport and subsequent redistribution from leaf to root.^179^

Another method of application of nanofertilizers to plants is seed priming, where seeds may be previously treated with nanosuspensions, usually in an aqueous medium containing an agrochemical of interest, which, due to the small size of NPs, may penetrate the seed coats, reducing targeted adverse effects such as salinity, drought, and heavy metal stresses.^182^ Direct absorption of NPs in seeds can occur by entering the coat via parenchymatic intercellular spaces, accompanied by diffusion in the cotyledon.^183^ This has been shown, in recent studies, to be related to the ability of these particles to slowly release their carriers and activate key genes during germination, promoting certain stress resistances.^184^ Although seed priming shows promise, there are still a limited number of studies on that topic.

Previous studies suggest that, due to their physicochemical properties, Fe nanofertilisers can be internalized through the previously described pathways, without necessarily relying on the conventional Strategy I or Strategy II Fe uptake mechanisms described in Section 1.^185,186^ However, when applied directly to the soil, the nanoparticle cargo may be released into the rhizosphere before internalization, allowing the dissolved Fe species to be absorbed through these conventional uptake mechanisms.^187^ Building on advances in nanotechnology-based fertilization, a recent study evaluated the potential of [Fe­(dmpp)_3_]-loaded nanoparticles, based on a 3,4-HPO iron chelate, to alleviate iron deficiency chlorosis in *G. max* grown in calcareous soil.^188^ In this work, the uptake of polymeric NPs by seed coating and the effect of encapsulated [Fe­(dmpp)_3_] on morphological and genetic expression factors have been analyzed. The results have shown that plants whose seeds were treated with the 20 μM nanosuspension grew, on average, 2.5 days faster up to V1 and 2.8 days up to V3, it also had 26% higher SPAD values at this stage. Roots and shoots of plants that underwent this treatment had 39% and 26% more fresh weight, respectively, and *IRT1* and *FERRITIN* gene expression increased 2.25- and 3.37-fold, respectively, when compared to plants treated with water. Plants whose seeds were treated with 10 μM nanosuspension had a 3.31-fold increase in *FERRITIN* gene expression compared with the solution of the same concentration.^188^ This study reveals the potential of polymeric NPs in providing a more sustainable delivery method than regular fertilizer application, with impact on both morphological and genetic expression factors. This study, together with other promising works, highlights the potential of nanoparticles (NPs) particularly for 3,4-HPO delivery.

Similarly, nano Fe and Mn (hydr)­oxides were reported as efficient micronutrient carriers and interact with iron chelates (such as Fe-EDDHA) to enhance Fe/Mn uptake and root accumulation.^189^ Also, biobased humic nanocomposites (iron-humic nanofertilizers), which utilize natural biopolymer matrices (humic substances combined with ferrihydrite), were used to provide controlled, long-term Fe availability in calcareous soils comparable to Fe-EDDHA.^190^ Biopolymer-based nanocarriers, including chitosan^191^ and lignosulfonate complexes,^192^ have also emerged as effective in providing controlled micronutrient uptake by plants. Other studies considering nanofertilisation strategies are summarized in Table 2.

**Table 2:** Studies Using NPs in Plants^a^

NP Type	NP Matrix	Load	Tested Plant Species	Treatment Application	Application Type	Effects	Reference
Carbon nanofiber	Carbon	Copper	Cicer arietinum	Seed Priming	Fertilizer	Nanoparticles increased the water uptake capacity, protein and total chlorophyll contents, shoot and root lengths, and germination rate,	^193,194^
Inorganic	Zinc Oxide	N/A	Pisum sativum	Soil Application	Fertilizer	Shoot height and dry weight increased with NP concentration. Root height and dry weight were largest at medium doses of NPs. The maximum chlorophyll content, as well as the maximum zinc levels in root, shoot, and grain, were achieved with the largest concentration of NPs. Magnesium, copper, and iron were also increased. Yield increased with NP concentration.	^195^
Metal-oxide	Zinc and Iron Oxides	N/A	Lactuca sativa	Foliar Application	Fertilizer	At maximum concentration, both NPs increased plant heights, leaf areas, fresh and dry weights of both roots and shoots.	^196^
Polymeric	Alginate-chitosan	GA_3_	Solanum lycopersicum	Seed priming	Growth promoter	Short-term increase in shoot length and dry weight, as well as an increase in root dry weight, when in low doses. In the long term, shoot lengths were increased both at low and high concentrations. These changes were significant even when compared to nonencapsulated GA_3_. At most concentrations, plants treated with GA_3_ NPs produced more fruit than the ones treated with nonencapsulated GA_3_.	^197^
Polymeric	Chitosan	Zinc Oxide	Zea mays	Foliar Application	Fertilizer	Grain yield was increased in plants treated with NPs. After 60 days, plant growth and SPAD levels were higher in treated plants.	^198^
Polymeric	Zein	N/A	Glycine max	Applied Hydroponically	Potential Agrochemical Delivery System	High doses increased leaf chlorophyll in early stages, and biomass in roots. Stems had reduced size in later stages. There was an increase in sodium, zinc, iron, and copper concentrations in treated roots, and a decrease in aluminum and magnesium at higher doses. Mild oxidative stress was detected. Requires further testing with encapsulated agrochemical.	^194^
Polymeric	PEG	Iron Oxide	Sorghum bicolor	Soil or Foliar Application	Fertilizer	In both applications, monodisperse iron oxide NPs treatment increased total chlorophyll, Zn and Fe content of shoots and total root Cu and Fe.	^199^
Polymeric	PLGA	Fe(dmpp)_3_	Glycine max	Seed priming	Fertilizer	Plants treated with 20 μM nanosuspension reached stage V3 faster and had higher SPAD levels at this stage. Root and shoot fresh weight were significantly increased. Expression of key genes *IRT1* and *FERRITIN* was significantly increased. Plants treated with 10 μM nanosuspension had a higher *FERRITIN* expression than those treated with nonencapsulated Fe(dmpp)_3_ at the same concentration.	^188^
Liposomes	Lipids	Selenium	Triticum aestivum	Foliar Application	Fertilizer	Liposomes successfully enriched the content of selenium without affecting biomass, carotenoids, chlorophylls, and the content of other minerals.	^200^

a N/A—not applicable. PLGA—Poly lactic-*co*-glycolic acid. PEG—Poly­(ethylene glycol).

## 6 Environmental Impact, Safety, and Future Perspectives

Although these chelates of the 3,4-HPO family appear to be more efficient at delivering Fe than the commercially available ones, additional studies are still required to fully assess their environmental fate. Particular attention should be given to their degradation pathways in soil, Fe release dynamics, mobility and leaching potential, interactions with soil microorganisms, and accumulation in edible plant tissues.^106^ While available evidence suggests lower environmental persistence than conventional synthetic chelators,^148,149^ a comprehensive environmental risk assessment is still needed before large-scale agricultural implementation.

Similarly, while the use of nanofertilizers is an encouraging strategy to address plant nutritional problems, their potential bioaccumulation inside plant tissues and subsequent entry into the food chain raises concerns about their long-term environmental impact.^168,201^ In addition, issues related to trophic transfer through food chains and long-term impacts on soil ecosystems should be considered when assessing the sustainability of nanoparticle-based fertilization strategies.^168,201,202^ Health concerns arise mainly from their small size and high surface reactivity, which enable them to bypass biological barriers and access cells, organs, and tissues, potentially causing toxic effects.^203^ Furthermore, improper management and application can lead to the accumulation of these particles in the environment, influencing soil microorganism composition, activity, and nutrient turnover.^202^ Consequently, the biodegradability of nanoformulations must also be investigated, even considering that the design 3,4-HPO Fe-chelate-loaded nanoparticles were made considering a biodegradable and biocompatible polymer for their production.

Despite the very good results on the application of 3,4-HPO Fe chelates as fertilizers, these complexes have been studied only in soybean crops. Thus, in future studies, it is crucial to investigate their use and efficacy in a wider range of crops, while optimizing soil, foliar, and fertigation strategies, and enhancing their role in improving crop nutritional quality and micronutrient biofortification through more sustainable formulations. Moreover, variations on the 3,4-HPO structure must be considered, particularly to study the potential effect of a charged chelate on the fertilization of the crops. The mechanism of action must also be further investigated, and for this, the use of labeled chelates must be considered as a relevant strategy. By incorporating these new approaches with sustainable crop management practices, it is possible to enhance crop yield and nutritional quality for both food security and nutritional deficiency challenges in an ever-changing world. Finally, a life cycle assessment (LCA) should be performed for both 3,4-HPO chelates and nanofertilizers to comprehensively evaluate their environmental impacts throughout their entire life cycle, from raw material extraction and production to agricultural application and end-of-life, as demonstrated in previous studies.^204,205^

In conclusion, there is still a challenge to address iron deficiency in crops, aiming for the production of healthy plants to meet the pressing food needs of a growing world population. Traditional strategies of mitigation, such as the application of Fe-based fertilizers, are limited by negative environmental impacts and reduced efficiency. Most recently, synthetic Fe chelates, especially those belonging to the 3,4-HPO family, proved to be useful to amend IDC in soybean crops, with improved results compared to those of conventional chelates.

The potential of the 3,4-HPO family, particularly the [Fe­(mpp)_3_] complex, as a promising alternative to overcoming IDC in soybean is supported by both its physiological efficacy and chemical versatility. From a synthetic point of view, these ligands and their corresponding iron complexes are characterized by relatively simple, straightforward, and sustainable syntheses. This synthetic route is highly versatile, allowing for diverse structural modifications to fine-tune the compounds’ properties. Agronomically, studies have shown that 3,4-HPO chelates enhance chlorophyll synthesis, promote overall plant biomass, and significantly increase iron accumulation and translocation from roots to shoots compared with traditional commercial chelates such as FeEDDHA. These combined chemical and physiological advantages position 3,4-HPO chelates as an efficient and sustainable solution for IDC management in soybean and eventually many other crops.

## 7 Literature Search Strategy

This review is based on a nonsystematic literature search. Relevant articles were identified through searches in PubMed, Web of Science, Scopus, and Google Scholar using combinations of keywords related to topics addressed in the paper. Additional publications were identified by screening the reference lists of the selected articles. Priority was given to peer-reviewed articles published in English, particularly recent studies considered to be relevant to the scope of this review. As this is a narrative review, no formal systematic review protocol or predefined inclusion/exclusion criteria were applied.
